# Grading der Tumorregression gastrointestinaler Karzinome nach neoadjuvanter Therapie

**DOI:** 10.1007/s00292-021-01041-5

**Published:** 2021-12-23

**Authors:** Drolaiz Liu, Rupert Langer

**Affiliations:** 1 Klinisches Institut für Pathologie und Molekularpathologie, Kepler Universitätsklinikum und Johannes Kepler Universität, Krankenhausstraße 9, 4021 Linz, Österreich; 2 Department of Pathology, GROW School for Oncology and Developmental Biology, Maastricht University Medical Center+, Maastricht, Niederlande

**Keywords:** Gastrointestinale Neoplasien, Histopathologie, Tumorregressionsgrading, Observervariabilität, Referenzstandards, Gastrointestinal neoplasms, Histopathology, Tumor regression grading, Observer variation, Reference standards

## Abstract

Prä- oder perioperative Chemo- oder Radiochemotherapie und anschließende Resektion ist die Standardtherapie von lokal fortgeschrittenem Ösophagus‑, Magen- und Rektumkarzinom. Eine Tumorregressionsgraduierung (TRG, auch Tumorregressionsgrad) kategorisiert das Ausmaß der regressiven Veränderungen nach neoadjuvanter Behandlung. Für gastrointestinale Karzinome existieren mehrere TRG-Systeme, die sich entweder auf das Ausmaß der therapieinduzierten Fibrose im Verhältnis zum Resttumor oder den geschätzten Anteil des Resttumors im Bereich des ehemaligen Tumorareals beziehen. Ein ideales TRG-System zeigt eine signifikante Interobserverübereinstimmung und bietet relevante prognostische Informationen – in den meisten Fällen ist eine vollständige oder nahezu vollständige Regression nach neoadjuvanter Therapie mit verbesserter Prognose verbunden. In diesem Review werden die am häufigsten verwendeten TRG-Systeme für gastrointestinale Karzinome vorgestellt und diskutiert. Zudem werden aktuelle Punkte wie die Standardisierung der Angabe von TRGs und die Thematik der Regression bei Lymphknotenmetastasen im Kontext eines TRG-Systems behandelt.

## Abstract (en)

Pre- or perioperative chemo- or radiochemotherapy and subsequent resection is the standard therapy for locally advanced esophageal, gastric, and rectal cancer. A tumor regression grading (TRG; also tumor regression grade) categorizes the extent of the regressive changes after a neoadjuvant treatment. There are several TRG systems for gastrointestinal carcinomas that relate either to the extent of the therapy-induced fibrosis in relation to the residual tumor or the estimated proportion of the residual tumor in the area of the former tumor area. An ideal TRG system shows significant interobserver agreement and offers relevant prognostic information – in most cases a complete or almost complete regression after neoadjuvant therapy is associated with an improved prognosis. In this review, the most commonly used TRG systems for gastrointestinal carcinomas are presented and discussed. In addition, current issues such as the standardization of TRG and the subject of regression in lymph node metastases in the context of a TRG system are discussed.

Eine prä- oder perioperative Therapie, typischerweise Chemo- oder Radiochemotherapie, gefolgt von der chirurgischen Resektion ist Standardtherapie für lokal fortgeschrittene gastrointestinale Neoplasien, insbesondere von Ösophagus‑, Magen- und Rektumkarzinomen. Dieses Vorgehen zeigte einen Überlebensvorteil für Patienten im Vergleich zu einer alleinigen Operation [4, 18]. Die Auswirkungen einer neoadjuvanten Therapie auf die Primärlokalisation des Tumors können durch makroskopische und insbesondere histopathologische Untersuchung der resezierten Präparate beurteilt werden [2].

In den letzten Jahrzehnten wurden mehrere Systeme für eine Tumorregressionsgraduierung (TRG, auch Tumorregressionsgrad) für Ösophagus‑, Magen- und Rektumkarzinome vorgeschlagen, die das Ausmaß der regressiven Veränderungen nach neoadjuvanter Therapie kategorisieren [2, 6, 17]. Der TRG liefert sehr wertvolle prognostische Informationen, da in den meisten Fällen eine vollständige oder subtotale Tumorregression nach neoadjuvanter Therapie mit besserer Prognose verbunden ist.

Im Folgenden werden die charakteristischen histopathologischen Befunde, die nach neoadjuvanter Therapie beobachtet werden, beschrieben und die Konzepte von TRG-Systemen, inklusive Beispiele einiger häufig verwendeter TRG-Systeme für Ösophagus‑, Magen- und Rektumkarzinome vorgestellt. Zudem werden Themen wie die Standardisierung der Angabe von TRGs und die Regression bei Lymphknotenmetastasen im Kontext eines TRG-Systems behandelt.

## Histopathologische Befunde nach neoadjuvanter Therapie

Der erste Schritt der pathologischen Abklärung ist die makroskopische Beurteilung der Resektionspräparate des Tumors. Hier ist eine erste grobe Abschätzung der Tumorregression möglich, aber noch wichtiger ist es, die Ausdehnung des sogenannten Tumorbetts (der früheren Lage des Tumors) abzuschätzen, um eine korrekte Einbettung für eine genaue histologische Untersuchung zu gewährleisten. Die Tumorregression nach neoadjuvanter Therapie stellt histologisch grundsätzlich eine subakute bis subchronische Entzündung nach zytotoxischer Wirkung dar, die wenige bis mehrere Wochen zuvor eingetreten ist. In den meisten Fällen werden die Tumoren mit einer gewissen Verzögerung nach Abschluss des letzten präoperativen Behandlungszyklus reseziert.

Bei vollständiger Tumorregression werden die Tumorzellen durch die Behandlung und/oder die nachfolgende Entzündungsreaktion zerstört und der Tumor durch fibröses oder fibroinflammatorisches Granulationsgewebe ersetzt. Im Gegensatz dazu kann ein Resttumor entweder reichlich vorhanden sein oder aber nur kleine Einzelzellen, tumorbudsähnliche Cluster oder Tumorzellgruppen umfassen. Tumoren können schrumpfen oder fragmentieren [8], sodass die Tumorregression auch einem zentrifugalen Muster folgen kann und der Resttumor nur in der oberflächlichen oder tiefen Peripherie des vorherigen Tumorareals und nicht im Tumorzentrum lokalisiert ist [2]. Resorptive Veränderungen umfassen eine histiozytäre Reaktion mit Schaumzellen oder auch mit Hämosiderin beladenen Makrophagen, Cholesterinspalten und Fremdkörperreaktionen sowie dystrophe Verkalkungen [2]. Bei neoadjuvant behandelten Adenokarzinomen können häufig muzinösen Veränderungen, gelegentlich mit ausgedehnten und teilweise azellulären Muzinseen beobachtet werden [2]. Dieses azelluläre Muzin sollte nicht als vitaler Resttumor betrachtet werden. Keines dieser histologischen Phänomene ist jedoch absolut spezifisch für eine therapieinduzierte Tumorregression und lässt auch per se nicht auf eine vorangegangene Therapie schließen. Anekdotisch ist es in der Routinepraxis auch vorgekommen, dass primär resezierten Tumoren ein Tumorregressionsgrad zugeordnet wurde. In diesem Zusammenhang sei jedoch erwähnt, dass das Vorhandensein von großflächigen Schaumzellaggregaten, ein eher zentrales Fibrosemuster und zytotoxische Gefäßveränderungen wie eine myxohyaline Intimaproliferation oft mit atypischen reaktiv veränderten Endothelzellen, Teleangiektasien, organisierenden Thromben und obliterierender Endarteritis, jedoch signifikant häufiger bei neoadjuvant behandelten Tumoren im Vergleich zu primär resezierten Tumoren beobachtet wird [2].

Auf zellulärer Ebene können die residuellen Tumorzellen ein im Vergleich zu unbehandelten Tumoren (oder auch der korrespondierenden prätherapeutischen Biopsie) oftmals weites, eosinophiles Zytoplasma mit Vakuolisierung oder onkozytärer Differenzierung aufweisen. Nukleäre Atypien wie Hyperchromasie, Pyknose, Karyorrhexis oder die Bildung großer, bizarrer Kerne sind ebenfalls häufige Befunde. Mitosen werden eher selten beobachtet. Diese Veränderungen können innerhalb von Tumoren auch heterogen ausgeprägt sein, sodass histologisch unauffällige Bereiche von Tumorinfiltrationen direkt neben derartig atypischen Drüsen oder Zellen mit signifikanten zytologischen Veränderungen beobachtet werden können [2]. Zudem zeigen sowohl das tumorale als auch das nichttumorale Stroma charakteristische Veränderungen, oftmals mit sogenannten bizarren Stromafibroblasten. Auch in nichtneoplastischem Nachbargewebe können therapieassoziierte Veränderungen wie Ödem und Entzündung beobachtet werden. Veränderungen in nichtneoplastischen Epithelien können denen von Tumorzellen ähneln (z. B. nukleärer Pleomorphismus, kondensiertes Chromatin und Eosinophilie). Solche Veränderungen von nichtneoplastischem Gewebe erscheinen gelegentlich besorgniserregend und können Schwierigkeiten in der Abgrenzung zu Karzinomen verursachen. Ein diagnostischer Fallstrick sind beispielsweise Atrophie und metaplastische Veränderungen nichtneoplastischer Drüsenstrukturen des Ösophagus und des Magens [2].

## Klassifikation der Tumorregression

**Figure 1:**
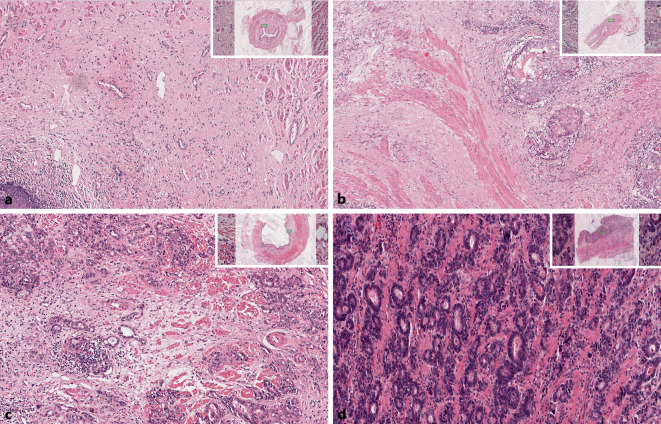

TRG-Systeme zielen darauf ab, das Ausmaß der regressiven Veränderungen nach zytotoxischer Behandlung zu kategorisieren, um potenzielle prognostische Informationen basierend auf objektiv bestimmbaren histopathologischen Befunden aufzuzeigen. Auf deskriptiver Ebene können Tumoren eine vollständige Regression oder verschiedene Mengen an Resttumor aufweisen: einige verstreute Resttumorzellen oder Gruppen innerhalb einer narbiger Fibrose („subtotale Regression oder fast komplette Regression“), mehr als nur einzelne residuelle Tumorzellen oder Tumorzellgruppen („partielle Regression“) oder eine signifikante Tumormenge mit oder ohne Anzeichen regressiver Veränderungen („keine signifikante Regression“; Abb. 1). Wie im Abschnitt „Histopathologische Befunde nach neoadjuvanter Therapie“ beschrieben, sind viele histopathologische Veränderungen nicht ganz spezifisch für eine Tumorregression. Daher beziehen sich die TRG-Systeme hauptsächlich auf einzelne, besser reproduzierbare Parameter.

Zur Klassifizierung des Tumorregressionsgrades lassen sich 2 Konzepte unterscheiden: einerseits die quantitative Abschätzung des Residualtumors (in Prozent oder deskriptiv), andererseits die Abschätzung des Verhältnisses zwischen Resttumor und regressiver Fibrose, die typischerweise auf einer Beschreibung basiert. Die TRG-Systeme nach Mandard [17] oder Dworak [6] sind Beispiele für TRG-Systeme, die sich auf das Verhältnis Tumor/Fibrose beziehen. Das Becker-System [2] oder das Kölner System [26] sowie das AJCC/CAP-System, das eine modifizierte Version des Ryan-Systems [25] darstellt, hingegen verwenden die Menge des Residualtumors bezogen auf das ursprüngliche Tumorareal. Eine Übersicht über verschiedenen TRG-Systeme und deren Kategorien geben Tab. 1 und 2.

**Table 1:** 

Deskriptive Regression	Mandard	AJCC	Becker
**Komplett**	*TRG 1*	*TRG 0*	*TRG 1a*
	Keine residuellen Tumorzellen	Keine residuellen Tumorzellen	Komplette Regression
**Subtotal**	*TRG 2*	*TRG 1*	*TRG 1b*
	Wenige Tumorzellen	Einzelzellen oder kleine Tumorzellgruppen	< 10 % Residualtumor
**Partiell**	*TRG 3*	*TRG 2*	*TRG 2*
	Mehr Fibrose als Residualtumor	Residualtumor mit desmoplastischer Reaktion	10–50 % Residualtumor
**Keine Regression**	*TRG 4*	*TRG 3* Minimale Regression	*TRG 3* > 50 % Residualtumor
	Mehr Residualtumor als Fibrose		
	*TRG 5*		
	Keine Regression		

*TRG* Tumorregressionsgraduierung

**Table 2:** 

Deskriptive Regression	Dworak	Köln
**Keine Regression**	*TRG 0*	*TRG 1*
	Keine Regression	> 50 % vitale residuelle Tumorzellen
**Partiell**	*TRG 1*	–
	Prädominanz von Tumorzellen mit signifikanter Fibrose und/oder Vaskulopathie	
	*TRG 2*	*TRG 2*
	Prädominanz der Fibrose mit vereinzelten Tumorzellen (leicht histologisch zu sehen)	10–50 % vitale residuelle Tumorzellen
**Subtotal**	*TRG 3*	*TRG 3*
	Nur ganz vereinzelt Tumorzellen in der Fibrose mit oder ohne azellulärem Muzin	< 10 % vitale residuelle Tumorzellen
**Komplett**	*TRG 4*	*TRG 4*
	Keine vitalen Tumorzellen	Komplette Regression

*TRG* Tumorregressionsgraduierung

## Prognostische Signifikanz der Tumorregression

Zahlreiche Studien haben die prognostische Bedeutung des TRG untersucht. Die stärkste Evidenz für den Zusammenhang zwischen TRG und Patientenergebnis wurde bei Karzinomen des oberen Gastrointestinaltrakts beobachtet. Eine komplette Tumorregression ist im Allgemeinen mit einer sehr guten Prognose assoziiert [1, 13]. Bei Magenkarzinomen wurde gezeigt, dass die prognostische Relevanz einer kompletten und subtotalen Regression (d. h. Mandard 1 und 2; Becker 1a und 1b) vergleichbar ist, sodass diese PatientInnen oft als „Responder“ bezeichnet werden [1]. Im Gegensatz dazu gibt es Daten für das Plattenepithel- und Adenokarzinom des Ösophagus, die nahelegen, dass bei diesen Tumoren eine subtotale und partielle Regression mit einer intermediären Prognose assoziiert ist [13]. Es ist daher nicht angebracht, die verschiedenen TRG-Systeme zu einem vereinfachten zweistufigen Klassifikationsschema mit „Respondern“ und „Nichtrespondern“ zu vereinfachen. Abhängig von der Fallzusammensetzung und den verwendeten statistischen Modellen waren TRG-Systeme neben dem Vorliegen von Lymphknotenmetastasen auch unabhängige prognostische Marker für das Überleben von Magen- und Ösophaguskarzinomen [1, 13], während in anderen Studien der Nachweis positiver Lymphknoten der einzige unabhängige Prognosefaktor war [5, 24]. Interessanterweise schnitt die ypT-Kategorie nach AJCC/UICC-Klassifikation häufig schlechter ab als die TRG- und yN-Kategorien [13, 24]. Im Hinblick auf das Regressionsmuster würde dies dafür sprechen, dass das Schrumpfen eines Tumors prognostisch nicht relevanter ist als eine Fragmentierung, wie für das Rektumkarzinom in einer Studie beschrieben [8]. Die gängigen Regressionsgraduierungssysteme berücksichtigen jedoch diese Unterschiede nicht.

Beim Rektumkarzinom sind die Daten zur klinischen Relevanz des TRG weniger aussagekräftig. Während eine vollständige Tumorregression konstant mit einem besseren krankheitsfreien und Gesamtüberleben assoziiert ist [16, 18], ist die Bedeutung einer subtotalen und partiellen Tumorregression weniger klar. Einige Kohortenstudien haben einen deutlichen prognostischen Einfluss einer partiellen Regression gezeigt [11, 16, 28], während andere Studien diese Ergebnisse nicht bestätigen [27]. Im Gegensatz zu Neoplasien des oberen Gastrointestinaltrakts erschwert beim Rektumkarzinom auch die hohe Variabilität verschiedener Behandlungsprotokolle die Vergleichbarkeit der Studien. Die wichtigsten prognostischen Faktoren für Rektumkarzinome mit oder ohne neoadjuvante Therapie sind der Status des zirkumferenziellen Resektionsrandes und – wie im oberen Gastrointestinaltrakt – das Vorhandensein oder Fehlen von Lymphknotenmetastasen. Da die Tumorregression jedoch stark mit diesen Faktoren assoziiert ist, ist erklärbar, warum der TRG in einigen Berechnungsmodellen die statistische Unabhängigkeit verliert [14].

## Tumorregression bei Lymphknotenmetastasen

Derzeit beziehen sich die TRG-Systeme auf regressive Veränderungen im primären Tumorareal („Tumorlager“ oder „Tumorbett“) und nicht auf Befunde in Lymphknoten oder Fernmetastasen. Eine Tumorregression kann jedoch auch in Lymphknoten, die metastasiert sind oder waren, beobachtet werden. Die histopathologischen Muster sind mit denen vergleichbar, die an primären Lokalisationen der Tumoren beobachtet wurden, wie z. B. Fibrose, resorptive Veränderungen, einschließlich histiozytärer Infiltrationen, und Fremdkörperreaktionen. Regressive und fibrotische Veränderungen, insbesondere in den mediastinalen Lymphknoten, können jedoch unabhängig von einer zytotoxischen Behandlung auftreten. Im Gegensatz dazu können kleine oder Mikrometastasen in Lymphknoten ohne signifikante Narbenbildung zurückgehen und der sichere Nachweis kleiner Metastasen vor der Behandlung ist mit bildgebenden Verfahren noch schwierig. Das Vorliegen von Lymphknotenmetastasen ist jedoch einer der wichtigsten prognostischen Faktoren bei gastrointestinalen Karzinomen nach neoadjuvanter Therapie und Operation [1, 7, 23]. Daher sollte bei Vorliegen einer Fibrose ohne Residualtumor in den ersten Schnittstufen eine weitere Aufarbeitung in Stufenschnitten in Betracht durchgeführt werden. Obwohl eine Graduierung der Tumorregression in Lymphknoten üblicherweise nicht durchgeführt wird, wurde vorgeschlagen, das Vorliegen oder Fehlen von Lymphknotenmetastasen unabhängig von regressiven Veränderungen in prognostische Stagingsysteme einzubeziehen [13]. Viele PathologInnen erwähnen in ihren Berichten ohnehin das Vorhandensein regressiver Veränderungen der Lymphknoten oder Lymphknotenmetastasen auch ohne weitere Regressionsgraduierung [31]. Bei Tumoren des oberen Gastrointestinaltraktes wurde gezeigt, dass eine komplette Regression in Lymphknotenmetastasen auch prognostisch einem Downstaging entspricht mit vergleichbaren Überlebensdaten wie bei ypN0-Tumoren ohne Regression, d. h. bereits prätherapeutisch negativem Lymphknotenstatus [21, 23]. Das Interobserveragreement für die Bewertung der Regression in Lymphknotenmetastasen wurde als substanziell beschrieben [23, 30]. Daten über die prognostische Bedeutung von regressiven Veränderungen an Lymphknoten mit noch nachweisbarem Residualtumor sind jedoch nicht konklusiv, sodass diesbezüglich weitere Studien nötig sind.

## Kritische Punkte der TRG

Ein kritischer Punkt bei der Bestimmung eines TRG ist die Inter- und Intraobservervariabilität. Je nach Studiendesign wurde die Interobservervariabilität einer TRG als nicht zufriedenstellend [3] oder als substanziell beschrieben [10]. In Einzel- und Grenzfällen kann eine exakte Zuordnung zu einem TRG schwierig sein. In der täglichen Praxis sind die meisten Tumore jedoch klar einem bestimmten TRG zuordenbar [10, 12, 28]. Eine Quantifizierung des Residualtumors mittels digitaler Bildanalyse könnte hierbei hilfreich sein [20]. Derartige Techniken könnten auch dazu beitragen, andere morphologische Parameter wie die reine Bestimmung des Residualtumors als prognostisch relevant zu identifizieren, um diese dann in ein noch valideres Regressionsgraduierungssystem aufzunehmen. Hinsichtlich der Aufarbeitung hat sich gezeigt, dass die Bewertung des gesamten Tumorareals, wie sie in der Praxis durchgeführt wird, eine bessere Interobserverübereinstimmung aufweist, als nur die Bewertung eines repräsentativen Schnittes (wie es in einigen Interobserverstudien gemacht wurde) [3, 10].

Ein anderer kritischer Punkt ist die immer noch fehlende Standardisierung bei der histologischen Aufarbeitung und der abschließenden Verwendung der TRG-Systeme. Zwar wird in der Praxis das Tumorlagers zumindest bis zu einer gewissen Größe komplett eingebettet [31], allgemein gültige Standards existieren aber diesbezüglich nicht. In der Erstbeschreibung wurde zumindest für das Becker-, das Mandard- und das Ryan-System eine komplette Aufarbeitung des Tumorlagers beschrieben [2, 17, 22]. Hinsichtlich der Verwendung von TRG-Systemen existieren weltweit große regionale Unterschiede. Während in englischsprachigen Ländern, insbesondere den USA und Großbritannien, sehr homogen ein von der AJCC und der CAP auch empfohlenes modifiziertes Ryan-System für alle Tumorentitäten verwendet wird, wird in Europa neben dem AJCC/CAP System auch das Mandard-System, das Becker-System (insbesondere für Tumoren des oberen Gastrointestinaltraktes) und das Dworak-System (für Rektumkarzinome, insbesondere in deutschsprachigen Ländern) angewendet. In Japan werden die dortigen Richtlinien eingesetzt, die eine Quantifizierung mit anderen Schwellenwerten als z. B. beim Becker-System beinhalten [31]. Generell wird jedoch ein vierstufiges System von vielen Experten als ideal angesehen [19, 29, 31]. Im Gegensatz zur AJCC-TNM-Klassifikation, wo die Angabe eines TRG zumindest für das Rektumkarzinom empfohlen wird, ist in der UICC-TNM-Klassifikation die Tumorregression nicht als relevanter Parameter aufgeführt. Die kürzlich veröffentlichen Datensätze für standardisierte Tumorbefunde der International Collaboration of Cancer Reporting (ICCR) empfehlen ebenfalls nur für das Rektumkarzinom eine bestimmten TRG (die AJCC/CAP-TRG), während für das Ösophaguskarzinom die 3 am häufigsten verwendeten TRGs (Mandard, Becker, AJCC/CAP) als Möglichkeiten vorgestellt werden. Beim Magenkarzinom wird in einem Kommentar auf diese Varianten eingegangen, im Haupttext jedoch ebenfalls das AJCC/CAP-System empfohlen [9].

## Schlussfolgerung und Ausblick

Die Tumorregressionsgraduierung ist als morphologischer und prognostischer Parameter in der pathologischen Aufarbeitung und Befundung von Resektaten von Tumoren des Gastrointestinaltraktes nach neoadjuvanter Therapie fest etabliert. Historisch und geografisch bedingt existieren eine Reihe von fast gleichwertigen Systemen, wobei eine verbindliche Empfehlung darüber, welches System verwendet werden sollte (und auch für welche Tumore), noch aussteht. Es ist deshalb sinnvoll, bei der Angabe eines Regressionsgrades den Namen des verwendeten Systems und im Idealfall auch die wörtliche Bedeutung anzugeben, um Fehlinterpretationen in der klinischen und wissenschaftlichen Umsetzung zu vermeiden. Eine internationale Standardisierung der Bewertung der TRG ist jedoch nach wie vor wünschenswert.

In den letzten Jahren wurden neben der Weiterentwicklung der konventionellen Radiochemotherapie- und Chemotherapieschemata für gastrointestinale Tumoren auch neue Therapieansätze für Tumorbehandlungen vorgestellt, wie zum Beispiel die zielgerichtete Anti-HER2-Therapie bei Adenokarzinomen des oberen Gastrointestinaltrakts oder zuletzt die Einführung der Immuncheckpoint-Inhibition [15]. Über die Gewebeveränderungen, die sich aus diesen neuen Behandlungsstrategien ergeben, ist jedoch wenig bekannt. Eine sorgfältige histopathologische Untersuchung des posttherapeutischen Gewebes kann – wie bei den initialen Beschreibungen der verschiedenen Regressionsgraduierungen – somit wichtige Aspekte der Wirkungen und Resistenzmechanismen dieser neuen Medikamente liefern. Inwieweit sich dies auf eine Regressionsgraduierung für künftig zu erwartenden Therapien auswirken wird, muss sorgfältig erarbeitet werden.

## Fazit für die Praxis

- Die makroskopische und histopathologische Aufarbeitung von Resektaten gastrointestinaler Karzinome nach neoadjuvanter Therapie sollte standardisiert durchgeführt werden mit einer im Idealfall kompletten Untersuchung des ursprünglichen Tumorareals.
- Die Angabe eines Tumorregressionsgrades im histopathologischen Befund wird empfohlen.
- Es existieren verschiedene, prognostisch fast gleichwertige Systeme für die Tumorregressionsgraduierung, wobei es aktuell noch keine fest verbindliche Empfehlung für die Verwendung eines speziellen Systems gibt.
- Die Rolle der Regression in Lymphknoten muss noch weiter untersucht werden.
